# Circulating Adiponectin Levels Are Inversely Associated with Mortality and Respiratory Failure in Patients Hospitalized with COVID-19

**DOI:** 10.1155/2023/4427873

**Published:** 2023-03-14

**Authors:** Bettina Hindsberger, Birgitte Lindegaard, Liv Rabøl Andersen, Simone Bastrup Israelsen, Lise Pedersen, Pal Bela Szecsi, Thomas Benfield

**Affiliations:** ^1^Center of Clinical Research and Disruption of Infectious Diseases (CREDID), Department of Infectious Diseases, Copenhagen University Hospital-Amager and Hvidovre, 2650 Hvidovre, Denmark; ^2^Department of Infectious Diseases, Copenhagen University Hospital–North Zealand, 3400 Hilleroed, Denmark; ^3^Department of Clinical Medicine, Faculty of Health and Medical Sciences, University of Copenhagen, 2200 Copenhagen, Denmark; ^4^Department of Clinical Biochemistry, Holbaek Hospital, 4300 Holbaek, Denmark

## Abstract

**Background:**

Chronic low-grade inflammation associated with a dysregulated adipose tissue might contribute to amplifying the inflammatory response in severe COVID-19. The aim of this study was to examine the association between levels of circulating leptin and adiponectin and the severity and mortality of COVID-19.

**Methods:**

Serum levels of leptin and adiponectin were determined at admission in 123 individuals with confirmed COVID-19 and their association with 90-day mortality and respiratory failure was analyzed by logistic regression analysis and expressed as odds ratios (ORs) with 95% confidence intervals (CIs).

**Results:**

The median values of circulating leptin and adiponectin were 7.2 ng/mL (IQR 3.8–13.4) and 9.0 *μ*g/mL (IQR 5.7–14.6), respectively. After adjustment for age, sex, body mass index, hypertension, diabetes, chronic obstructive pulmonary disease, and oxygen saturation at admission, a doubling of circulating adiponectin was associated with a 38% reduction in odds of 90-day mortality (OR 0.62, CI 0.43–0.89) and a 40% reduction in odds of respiratory failure (OR 0.60, CI 0.42–0.86). The association tended to be strongest in individuals below the median age of 72 years. Circulating leptin was not associated with outcomes.

**Conclusions:**

Circulating adiponectin at admission was inversely associated with mortality and respiratory failure in SARS-CoV-2 infection. Further studies are needed to elucidate how exactly adipokines, especially adiponectin, are linked to the progression and prognosis of COVID-19.

## 1. Background

While most cases of COVID-19 are mild, some individuals suffer from more severe forms of the disease. They are at risk of developing pneumonia, acute respiratory distress syndrome (ARDS), and viral sepsis, which may require intensive care and mechanical ventilation [1]. The mechanisms underlying the pathogenesis of severe COVID-19 are still not fully understood, but hyperinflammation plays an important part [2]. Furthermore, visceral adiposity, obesity, and obesity-associated conditions such as diabetes and cardiovascular disease have been found to increase the risk of severe disease and mortality in COVID-19 [3, 4]. It has been suggested that the chronic low-grade inflammation associated with a dysregulated adipose tissue might contribute by amplifying the inflammatory response [5].

In the past decades, it has become evident that adipose tissue is not merely an energy store, but rather a physiologically active tissue involved in the regulation of both endocrine and immune processes. These functions are mediated by the production of soluble factors termed adipokines, of which leptin and adiponectin are the most abundant [6]. Leptin functions as a key regulator of body weight and appetite, and circulating leptin levels are proportional to the amount of white adipose tissue [7]. It also exerts proinflammatory effects, and thus high leptin levels have been linked to autoimmune diseases, while low levels might increase susceptibility to infections [8]. Adiponectin regulates glucose and lipid metabolism and exhibits anti-inflammatory features. Low levels have been reported in unhealthy obesity, diabetes, coronary heart disease, and nonalcoholic fatty liver disease [9–12]. The role of these adipokines in acute illness is incompletely understood, but they might provide a link between dysfunctional adipose tissue and the exaggerated inflammatory response seen in severe COVID-19. Hence, this study aims to examine the association between circulating leptin and adiponectin and the severity and mortality of SARS-CoV-2 infection in a cohort of patients hospitalized with COVID-19.

## 2. Materials and Methods

### 2.1. Study Design

This retrospective study included adults aged 18 years or older with COVID-19 admitted to Copenhagen University Hospital–Amager and Hvidovre, Denmark, between March 10 and May 31, 2020. Details of the cohort have been described previously [13]. In brief, all consecutive individuals admitted were included. All cases were confirmed by SARS-CoV-2 reverse-transcriptase-polymerase-chain-reaction on an oropharyngeal swab or lower respiratory tract specimen. At the time of the study, the Wuhan COVID-19 variant was dominant. Remdesivir, dexamethasone, and anti-interleukin-6 treatment was not yet available. Data including patient characteristics (age, sex, body mass index (BMI), comorbidities, and treatment limits), vital parameters, and laboratory measurements were transferred from electronic health records and managed using Research Electronic Data Capture browser-based software (REDCap, Vanderbilt, TN, USA). Only patients with an available blood sample, drawn within four days from admission, were included in our study. Serum was separated by centrifugation and stored at minus 80°C. Individuals were followed for 90 days from sampling or until death, whichever occurred first.

### 2.2. Study Approval

The study was approved by the Danish Patient Safety Authority (record no. 31-1521-309) and the Regional Data Protection Center (record no. P-2020-492). Measurements of biomarkers in stored samples from the biobank were approved by the Ethical Committee of the Capital Region of Denmark (record no. H-20047597). A requirement of individual informed consent was exempted by the committee.

### 2.3. Outcomes

The primary outcome was 90-day mortality from the time of blood sampling. The secondary outcome was respiratory failure defined as receiving mechanical ventilation during admission.

### 2.4. Adiponectin and Leptin Measurements

Adiponectin and leptin levels in serum were determined by immunoassay with minor modifications according to the manufacturer's instructions using magnetic fluorescently labelled microsphere beads with suspension array system ProcartaPlex (EPX01A-12032-901 and EPX01A-12039-901) (ThermoFisher, Vienna, Austria) and analyzed on a BioPlex 200 (Bio-Rad, Hercules, CA, USA). For each of the analytes, the lower and upper limits of quantification were defined as the lowest/highest measurable standard ±three times the standard deviation. Values below or above these limits were assigned a value of 10% lower or higher than the limit of quantification.

### 2.5. Statistics

Descriptive statistics are presented as medians with interquartile ranges (IQRs) for continuous variables and numbers with percentages for categorical variables. The Mann–Whitney *U* test, Kruskal–Wallis test, *χ*^2^-test, and Fisher's exact test were used to compare groups, as appropriate. Correlations were estimated by Spearman's coefficient. Odds ratios (ORs) and 95% confidence intervals (CIs) associated with outcomes were estimated by logistic regression. ORs and corresponding 95% CIs were displayed in forest plots. Models were adjusted for age, sex, and BMI, as these variables were considered potential confounders. Further adjustment for confounding was performed after evaluating if any other variables were associated with circulating adipokine levels or outcome. Only variables available for >90% of the subjects were added to this fully adjusted model. An additional analysis on respiratory failure was performed, in which subjects with a do-not-intubate order were omitted. The non-normally distributed variables leptin, adiponectin, adiponectin/leptin ratio, and BMI were log2-transformed prior to analysis. Thus, the estimated OR corresponds to the OR associated with a doubling of circulating adiponectin and leptin levels. Age was modelled as categorical (≤60 years, 61–80 years, ≥81 years) as the relationship between age and mortality is nonlinear [4]. A two-tailed value of *p* < 0.05 was considered statistically significant. Statistics were performed in R version 4.0.3 [14].

## 3. Results

### 3.1. Study Population

We obtained adipokine measurements from 123 patients out of 324 patients hospitalized with COVID-19. The median time from admission to blood sampling was two days (IQR 2–3). Subjects had a median age of 72 years, slightly more were males, and they were slightly overweight (Table 1). The most common comorbidity was hypertension (48%) followed by diabetes (29%). At admission, most subjects presented with infiltration on chest X-ray, half received supplemental oxygen, and the majority had elevated levels of plasma lactate dehydrogenase (LDH) and C-reactive protein (CRP) (Table 1). When comparing our cohort with the overall cohort, subjects with or without available adipokine measurements were similar on many parameters, including age, BMI, and comorbidities (Supplementary Table 1). However, some differences were present as well. In the group with measured circulating adipokine levels, more were male, and they were more ill at admission, with a slightly higher respiratory rate, a slightly lower saturation, more need of supplemental oxygen, and higher levels of plasma alanine aminotransferase (ALT), LDH, and CRP (Supplementary Table 1).

### 3.2. Associations between Circulating Adipokine Levels and Clinical Characteristics

The median values of circulating leptin and adiponectin were 7.2 ng/mL (IQR 3.8–13.4) and 9.0 *μ*g/mL (IQR 5.7–14.6), respectively (Table 1). Levels of circulating leptin did not differ across the three age groups (*p* = 0.37), while levels of circulating adiponectin were found to be higher with increasing age (*p* < 0.001) (Table 2). Women had higher levels of circulating adipokines than men (leptin (12.2 ng/mL vs. 5.7 ng/mL) and adiponectin (12.5 *µ*g/mL vs. 8.0 *µ*g/mL)). In addition, levels of circulating adiponectin were affected in patients with certain comorbidities compared to patients without these comorbidities, namely, hypertension (10.6 *µ*g/mL vs. 7.6 *µ*g/mL), diabetes (7.2 *µ*g/mL vs. 9.4 *µ*g/mL), and chronic obstructive pulmonary disease (COPD) (14.8 *µ*g/mL vs. 8.9 *µ*g/mL) (Table 2). Circulating leptin was not affected by any comorbidity. In addition to the associations listed in Table 2, correlations between levels of circulating adipokines and BMI were assessed. Circulating leptin was positively correlated with BMI (*r* = 0.46, *p*<0.0001), while there was only a weak inverse correlation between BMI and circulating adiponectin (*r* = −0.20, *p*=0.04).

### 3.3. Association of Circulating Leptin and Adiponectin with 90-Day Mortality

At the 90-day followup, 37 patients (30%) had died. Generally, subjects who had died at followup were older and more likely to suffer from hypertension. At admission, they had lower saturation and blood lymphocyte counts as well as higher levels of plasma creatinine and LDH than patients alive at followup (Table 1). No differences in survival were found by sex, BMI, or any of the other included comorbidities, vital parameters, or laboratory findings. Nor were there any differences in levels of circulating leptin or adiponectin between survivors and nonsurvivors (Table 1).

**Figure 1:**
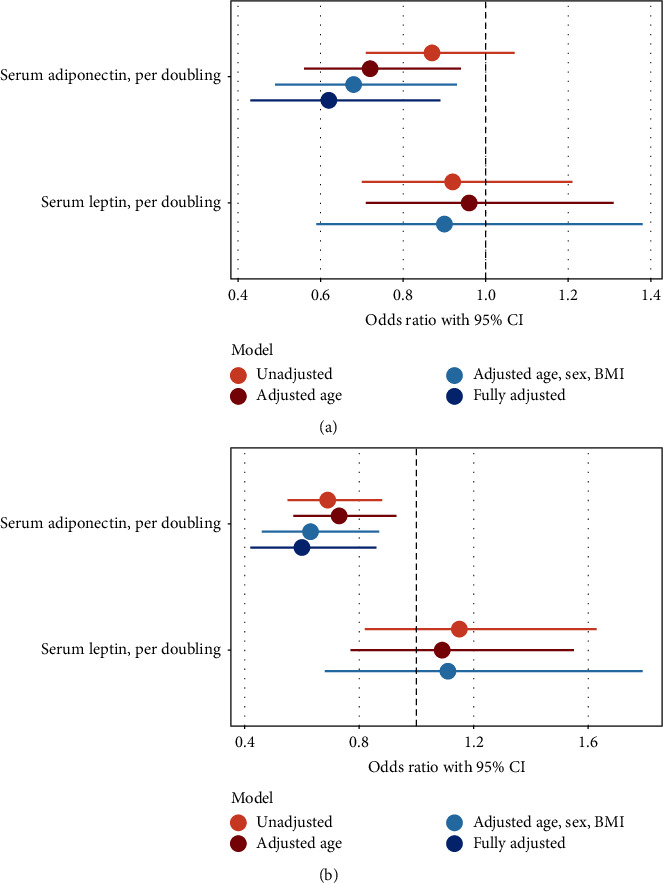
Forest plots of the association between adipokines and 90-day mortality (a) and respiratory failure (b) in patients hospitalized with COVID-19. Fully adjusted (only adiponectin): adjusted for age groups, sex, BMI, hypertension, diabetes, chronic obstructive pulmonary disease, and oxygen saturation at admission. As adiponectin and leptin were log2-transformed prior to analysis, the estimated odds ratio corresponds to the odds ratio associated with a doubling of circulating adiponectin and leptin levels. BMI: body mass index, CI: confidence intervals.

Circulating adipokine levels were not significantly associated with 90-day mortality in unadjusted logistic regression models (Figure 1(a)). However, adjusted for age groups, sex, and BMI, circulating adiponectin was associated with 90-day mortality (OR 0.68, CI 0.49–0.93). Thus, a doubling of adiponectin corresponded to a 32% decrease in the odds of 90-day mortality. Hypertension, diabetes, COPD, and oxygen saturation at admission were found to be potential confounders (Tables 1 and 2), and consequently, these comorbidities were included in a third analysis (Figure 1(a)). In this model, the association between circulating adiponectin and 90-day mortality remained (OR 0.62, CI 0.43–0.89). When evaluating the impact of the individual variables on the model, adjustment for age groups had the biggest effect (OR 0.72, CI 0.56–0.94), followed by adjustment for hypertension (OR 0.79, CI 0.63–0.99) (not shown).

**Figure 2:**
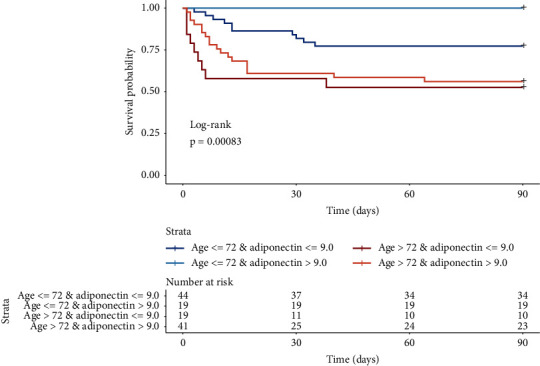
Cumulative estimate survival probability stratified by median age (72 years) and median adiponectin level (9.0 *μ*g/mL) of patients hospitalized with COVID-19. Statistics performed by log rank test for equal mortality in the four groups, as defined.

Because age correlated with adiponectin levels and was the strongest modifier of the association between circulating adiponectin and mortality, exploratory stratified survival curve analysis was performed by median age (72 years) and median level of circulating adiponectin (9.0 *μ*g/mL). The survival curve analysis illustrates that older age increases the risk of 90-day mortality independently of adiponectin levels (Figure 2). Among 19 individuals aged 72 years or younger, all were alive at the 90-day followup for individuals with circulating adiponectin above the median, while in the group with below median circulating adiponectin, 10 of 44 (23%) had died at follow-up (Figure 2). For individuals older than 72 years, levels of circulating adiponectin did not alter survival status at day 90.

Circulating leptin at admission was not associated with 90-day mortality in any of the adjusted models (Figure 1(a)).

### 3.4. Association of Circulating Leptin and Adiponectin with Respiratory Failure

Respiratory failure leading to mechanical ventilation had developed in 20 patients (16%) at the 90-day followup. Median levels of circulating adiponectin in subjects receiving mechanical ventilation compared to subjects not receiving mechanical ventilation were 6.4 *μ*g/mL (IQR 2.8–9.5) and 9.3 *μ*g/mL (IQR 6.1–16.3), respectively, (*p* < 0.01). For circulating leptin, the median in these two groups were 10.7 ng/mL (IQR 6.3–13.1) and 6.2 ng/mL (IQR 3.6–13.4) (*p*=0.28). Accordingly, circulating adiponectin was associated with respiratory failure in an unadjusted model (OR 0.69, CI 0.55–0.88) (Figure 1(b)). The association was stronger in a model adjusted for age groups, sex, and BMI (OR 0.63, CI 0.46–0.87), and when including comorbidities and oxygen saturation at admission (OR 0.60, CI 0.42–0.86). The analysis performed exclusively on subjects without a do-not-intubate order provided similar results for both the unadjusted model (OR 0.74, CI 0.57–0.96) and the fully adjusted model (OR 0.59, CI 0.40–0.87). Levels of circulating leptin at admission were not associated with respiratory failure in any of the models (Figure 1(b)).

### 3.5. Association of Adiponectin/Leptin Ratio with Mortality and Respiratory Failure

The adiponectin/leptin ratio was associated with respiratory failure in unadjusted analysis (OR 0.75, CI 0.61–0.91) and after adjustment for age, sex, and BMI (OR 0.69, CI 0.52–0.91). The adiponectin/leptin ratio was not associated with 90-day mortality in unadjusted analysis (OR 0.95, CI 0.80–1.11) or after adjustment for age, sex, and BMI (OR 0.81, CI 0.64–1.02).

## 4. Discussion

In our study, we found that higher circulating adiponectin at admission was associated with reduced odds of 90-day mortality and respiratory failure in patients hospitalized with COVID-19. Circulating leptin was not associated with 90-day mortality or respiratory failure in any of the models, while the adiponectin/leptin ratio was associated with respiratory failure but not mortality. Generally, levels of circulating adipokines varied between males and females, while only circulating adiponectin differed between age groups and between subjects with or without hypertension, diabetes, and COPD. Both circulating leptin and circulating adiponectin correlated with BMI, though the correlation between circulating leptin and BMI was stronger.

The associations between higher circulating adiponectin and reduced odds of 90-day mortality and respiratory failure were strengthened when adjusting for age, sex, BMI, hypertension, diabetes, COPD, and oxygen saturation at admission. Adjustment for age had the biggest individual effect, probably due to the strong association between age and circulating adiponectin. We observed higher levels of circulating adiponectin in older patients, consistent with previous reports on healthy subjects [15]. In their study, Obata and colleagues found that adiponectin was increased with age, independently of body fat status, glucose metabolism, and lipid profiles. This relationship might also explain, why subjects with hypertension were found to have significantly higher levels of adiponectin even though hypertension has been linked to low levels previously [16]. Furthermore, survival curve analysis performed by median age and median circulating adiponectin level showed that the effect of adiponectin levels on the risk of 90-day mortality appears to be more profound among younger individuals than older individuals. Likewise, for a range of comorbidities including cardiovascular disease, hypertension, and diabetes, it has been shown that the effect of the comorbidity on COVID-19 severity increases with young age [17]. Perhaps, the effect of one condition is less pronounced in the elderly population, as the occurrence of multiple chronic conditions, disability, and frailty is higher in this group than in the general population, suggesting an overall increased vulnerability to disease [18].

Our results indicate that adiponectin could be involved in the pathogenesis of severe COVID-19. Other studies on adiponectin and COVID-19 were mostly smaller and results were sometimes discrepant with ours. A single study of similar size but with a younger population with lower mortality assessed circulating adiponectin in relation to mortality or respiratory failure and was unable to show an association between circulating adiponectin and admission to intensive care units or in-hospital death in patients hospitalized with COVID-19 [19]. Two studies, however, showed an association between adiponectin and the severity of COVID-19 but did not assess outcomes in relation to adiponectin [20, 21]. Reiterer et al. compared levels of circulating adiponectin between groups of critically ill patients and found that circulating adiponectin in patients with COVID-19 ARDS was decreased by 50–60% compared to non-COVID-19 controls with and without ARDS [20]. Similarly, Kearns et al. found that circulating adiponectin was significantly lower in patients with COVID-19 ARDS compared to non-COVID-19 ARDS [21]. Other studies of limited size were unable to show any association between adiponectin levels and COVID-19 according to disease severity (mild, moderate, or severe), Sequential Organ Failure Assessment (SOFA) score, and type of respiratory support required [22–25]. Further studies on adiponectin's role in COVID-19 are warranted in order to confirm and refute our findings and to study adiponectin's role in populations treated with antivirals and anti-inflammatory agents.

As is the case for adiponectin, reports on the role of circulating leptin in SARS-CoV-2 infection are somewhat conflicting. Wang et al. showed that levels of circulating leptin were higher in patients with severe COVID-19 compared to milder cases, while Singh et al. found nonsurvivors to have higher levels than survivors [26, 27]. In addition, the study by Singh et al. showed a correlation between circulating leptin and SOFA score at baseline. Tonon et al. found higher circulating leptin levels in patients requiring noninvasive mechanical ventilation, but this association did not persist in multivariate regression models [25]. Other studies report no association between circulating leptin and severity or outcomes in SARS-CoV-2 infection [19, 22, 24, 28], in agreement with the results from our study. This suggests that leptin plays only a limited role in the pathogenesis of COVID-19.

Our study was strengthened by the consecutive enrollment of patients, the fairly large study size, and the complete followup on subjects. Given that blood samples were drawn within four days of admission, and since the study was carried out before any treatment regimens for COVID-19 were established, levels of circulating adipokines were unlikely to be affected by the administration of dexamethasone, tocilizumab, or other immunomodulatory therapy. Some limitations of the study should be considered. Association studies do not show causality and we cannot account for unmeasured residual confounding. Also, we only included one sample from each patient, and therefore the dynamics in adipokine levels could not be examined.

In conclusion, we found that higher circulating adiponectin was associated with reduced odds of mortality and respiratory failure in patients hospitalized with COVID-19. Further studies are needed to elucidate how adipokines, especially adiponectin, affect the progression and prognosis of COVID-19, also in the context of immunomodulatory treatment with corticosteroids and IL-6 inhibitors.

## Figures and Tables

**Table 1:** Characteristics, vital parameters, and laboratory findings at admission of patients hospitalized with COVID-19.

	Non-survivors (*n* = 37)	Survivors (*n* = 86)	Total (*n* = 123)	p value
Age				
≤60 years, *n* (%)	2 (5.4)	36 (41.9)	38 (30.9)	
61–80 years, *n* (%)	18 (48.6)	35 (40.7)	53 (43.1)	
≥81 years, *n* (%)	17 (45.9)	15 (17.4)	32 (26.0)	<0.0001
Age, median [IQR]	79 [71, 84]	65 [53, 76]	72 [59, 81]	<0.0001
Sex				
Female, *n* (%)	14 (37.8)	36 (41.9)	50 (40.7)	
Male, *n* (%)	23 (62.2)	50 (58.1)	73 (59.3)	0.83
BMI, median [IQR]^a^	27.4 [24.2, 31.1]	27.9 [24.3, 31.5]	27.9 [24.2, 31.2]	0.70
Hypertension, *n* (%)	25 (67.6)	34 (39.5)	59 (48.0)	<0.01
AMI and/or heart failure, *n* (%)	3 (8.1)	7 (8.1)	10 (8.1)	1.00
Diabetes, *n* (%)	15 (40.5)	21 (24.4)	36 (29.3)	0.11
COPD, *n* (%)	4 (10.8)	6 (7.0)	10 (8.1)	0.72
Asthma, *n* (%)	5 (13.5)	10 (11.6)	15 (12.2)	1.00
Infiltration on chest X-ray, *n* (%)	34 (91.9)	69 (80.2)	103 (83.7)	0.18
Findings at admission				
respiratory rate (breaths/min) median [IQR]^b^	22 [20, 30]	20 [18, 26]	21 [18, 28]	0.15
Oxygen saturation (%,) median [IQR]	94 [91, 96]	96 [94, 98]	95 [93, 97]	0.03
Supplemental oxygen, *n* (%)	22 (59.5)	39 (45.3)	61 (49.6)	0.22
Laboratory findings at admission				
Blood				
Lymphocytes × 10^9^/L, median [IQR]^c^	0.8 [0.6, 1.2]	1.0 [0.8, 1.3]	1.0 [0.7, 1.3]	0.05
Platelets × 10^9^/L, median [IQR]^c^	199 [166, 229]	200 [173, 264]	199 [168, 251]	0.34
Plasma				
Creatinine (*µ*mol/L), median [IQR]^c^	98 [85, 132]	84 [69, 97]	91 [75, 105]	<0.01
ALT (U/L), median [IQR]^c^	33 [28, 53]	32 [22, 56]	32 [24, 54]	0.29
LDH (U/L), median [IQR]^d^	406 [312, 523]	328 [237, 417]	336 [251, 456]	0.03
CRP (mg/L), median [IQR]^c^	110 [52, 183]	95 [50, 146]	98 [51, 153]	0.22
Serum				
Leptin (ng/mL), median [IQR]	7.2 [3.7, 12.7]	7.2 [3.8, 13.8]	7.2 [3.8, 13.4]	0.60
Adiponectin (*µ*g/mL), median [IQR]	8.9 [3.9, 14.5]	9.0 [6.0, 14.5]	9.0 [5.7, 14.6]	0.50
Adiponectin/leptin ratio, median [IQR]	1.5 [0.6, 2.4]	1.0 [0.5, 2.8]	1.1 [0.5, 2.6]	0.89

a: BMI was missing for 17 subjects. b: respiratory rate was missing for 1 subject. c: lymphocytes, platelets, creatinine, ALT and CRP were missing for 18 subjects. d: LDH was missing for 24 subjects. ALT: alanine aminotransferase; AMI: acute myocardial infarction; BMI: body mass index; COPD: chronic obstructive pulmonary disease; CRP: C-reactive protein; LDH: lactate dehydrogenase.

**Table 2:** Levels of circulating leptin and adiponectin in different subgroups of patients hospitalized with COVID-19.

	Leptin (ng/mL), median [IQR]	Adiponectin (*µ*g/mL), median [IQR]
Age		
≤60 years	8.2 [4.0, 12.4]	7.0 [4.5, 9.0]
61–80 years	7.8 [4.2, 13.9]	10.5 [5.6, 16.2]
≥81 years	6.0 [2.6, 13.3]	13.5 [7.3, 20.2]
p value	0.37	<0.001
Sex		
Female	12.2 [4.9, 22.6]	12.5 [6.2, 18.8]
Male	5.7 [2.9, 10.2]	8.0 [5.1, 10.8]
p value	<0.001	0.01
Hypertension		
Yes	7.4 [3.8, 12.9]	10.6 [6.9, 17.7]
No	6.2 [3.4, 14.3]	7.6 [4.3, 12.9]
p value	0.97	<0.01
AMI and/or heart failure		
Yes	6.8 [3.9, 11.8]	8.2 [3.7, 9.2]
No	7.2 [3.8, 13.5]	9.0 [5.7, 14.6]
p value	0.81	0.23
Diabetes		
Yes	6.9 [3.3, 12.8]	7.2 [3.8, 10.6]
No	7.2 [3.8, 14.0]	9.4 [6.1, 15.6]
p value	0.70	0.03
COPD		
Yes	4.6 [3.8, 15.9]	14.8 [11.5, 21.2]
No	7.3 [3.7, 13.0]	8.9 [5.0, 14.2]
p value	0.84	0.02
Asthma		
Yes	8.6 [5.6, 12.1]	10.2 [6.3, 15.7]
No	6.6 [3.4, 13.5]	8.9 [5.1, 14.5]
p value	0.48	0.51
Infiltration on chest X-ray		
Yes	7.1 [3.8, 13.5]	9.0 [5.1, 15.6]
No	8.1 [3.5, 13.0]	8.3 [6.1, 10.6]
p value	0.94	0.42
Supplemental oxygen at admission		
Yes	7.3 [4.5, 14.0]	9.2 [6.1, 14.4]
No	7.0 [2.8, 12.3]	8.5 [5.2, 14.6]
p value	0.21	0.93

AMI: acute myocardial infarction; COPD: chronic obstructive pulmonary disease.

## Data Availability

The data used to support the findings of this study are available from the corresponding author upon reasonable request.

## Supplementary Materials

Supplementary Materials Supplementary Table 1 provides information on characteristics, vital parameters, and laboratory findings at admission for the overall cohort of patients admitted with COVID-19 to Copenhagen University Hospital–Amager and Hvidovre, Denmark, between March 10 and May 31, 2020.
