# Supplementary material for: Circulating Adiponectin Levels Are Inversely Associated with Mortality and Respiratory Failure in Patients Hospitalized with COVID-19
Source: Int J Endocrinol. 2023 Mar 14;2023:4427873. doi: 10.1155/2023/4427873 (PMC10030212; doi:10.1155/2023/4427873)
Supplement: Supplementary Materials — Supplementary Table 1 provides information on characteristics, vital parameters, and laboratory findings at admission for the overall cohort of patients admitted with COVID-19 to Copenhagen University Hospital–Amager and Hvidovre, Denmark, between March 10 and May 31, 2020. [file 4427873.f1.docx]

**Supplementary Table 1. Characteristics, vital parameters, and laboratory findings at admission of patients in the overall cohort**

|  | Subjects with adipokine measurements (n = 123) | Subjects without adipokine measurements (n = 201) | Total (n = 324) | p-value |
| --- | --- | --- | --- | --- |
| Age |  |  |  |  |
| ≤ 60 years, n (%) | 38 (30.9) | 52 (25.9) | 90 (27.8) |  |
| 61-80 years, n (%) | 53 (43.1) | 91 (45.3) | 144 (44.4) |  |
| ≥ 81 years, n (%) | 32 (26.0) | 58 (28.9) | 90 (27.8) | 0.61 |
| Age, median [IQR] | 72 [59, 81] | 73 [58, 82] | 72 [58, 81] | 0.86 |
| Sex |  |  |  |  |
| Female, n (%) | 50 (40.7) | 109 (54.2) | 159 (49.1) |  |
| Male, n (%) | 73 (59.3) | 92 (45.8) | 165 (50.9) | 0.02 |
| BMI, median [IQR]^a^ | 27.9 [24.2, 31.2] | 26.5 [23.2, 31.8] | 27.2 [23.5, 31.6] | 0.29 |
| Hypertension, n (%) | 59 (48.0) | 84 (41.8) | 143 (44.1) | 0.33 |
| AMI and/or heart failure, n (%) | 10 (8.1) | 29 (14.4) | 39 (12.0) | 0.13 |
| Diabetes, n (%) | 36 (29.3) | 50 (24.9) | 86 (26.5) | 0.46 |
| COPD, n (%) | 10 (8.1) | 23 (11.4) | 33 (10.2) | 0.44 |
| Asthma, n (%) | 15 (12.2) | 15 (7.5) | 30 (9.3) | 0.22 |
| Infiltration on chest x-ray, n (%) | 103 (83.7) | 163 (81.1) | 266 (82.1) | 0.65 |
| Findings at admission |  |  |  |  |
| Respiratory rate/min, median [IQR]^b^ | 21 [18, 28] | 20 [18, 22] | 20 [18, 24] | < 0.01 |
| Oxygen saturation, %, median [IQR]^b^ | 95 [93, 97] | 96 [94, 98] | 96 [94, 98] | < 0.01 |
| Supplemental oxygen, n (%)^b^ | 61 (49.6) | 58 (29.9) | 119 (37.5) | < 0.001 |
| Laboratory findings at admission  Blood |  |  |  |  |
| Lymphocytes x 10^9^/L, median [IQR]^c^ | 1.0 [0.7, 1.3] | 1.0 [0.8, 1.5] | 1.0 [0.7, 1.4] | 0.26 |
| Platelets x 10^9^/L, median [IQR]^d^ | 199 [168, 251] | 197 [158, 246] | 199 [162, 248] | 0.48 |
| Plasma  Creatinine µmol/L, median [IQR]^e^ | 91 [75, 105] | 86 [65, 112] | 86 [68, 111] | 0.63 |
| ALT U/L, median [IQR]^f^ | 32 [24, 54] | 26 [18, 41] | 29 [20, 45] | < 0.01 |
| LDH U/L, median [IQR]^g^ | 336 [251, 456] | 251 [210, 337] | 278 [220, 383] | < 0.0001 |
| CRP mg/L, median [IQR]^h^ | 98 [51, 153] | 53 [24, 104] | 65 [35, 127] | < 0.0001 |

a: BMI was missing for 76 subjects; b: Respiratory rate, saturation and supplemental oxygen were missing for 7 subjects; c: Lymphocytes were missing for 46 subjects; d: Platelets were missing for 43 subjects; e: Creatinine was missing for 40 subjects; f: ALT was missing for 47 subjects; g: LDH was missing for 66 subjects; h: CRP was missing for 44 subjects.

ALT: alanine aminotransferase; AMI: acute myocardial infarction; BMI: body mass index; COPD: chronic obstructive pulmonary disease; CRP: C-reactive protein; LDH: lactate dehydrogenase.
